# Do children and adolescent ice hockey players with and without a history of concussion differ in robotic testing of sensory, motor and cognitive function?

**DOI:** 10.1186/s12984-016-0195-9

**Published:** 2016-10-12

**Authors:** C. Elaine Little, Carolyn Emery, Stephen H. Scott, Willem Meeuwisse, Luz Palacios-Derflingher, Sean P. Dukelow

**Affiliations:** 1 Faculty of Kinesiology, University of Calgary, Calgary, AB Canada; 2 Faculty of Kinesiology and Cumming School of Medicine, University of Calgary, Calgary, AB Canada; 3 Department of Biomedical and Molecular Sciences, Queen’s University, Kingston, ON Canada; 4 Faculty of Kinesiology, Cumming School of Medicine, and Community Health Sciences, University of Calgary, Calgary, AB Canada; 5 Department of Clinical Neurosciences, Cumming School of Medicine, University of Calgary, Calgary, AB Canada

**Keywords:** Robot, Sensorimotor, Cognitive assessment, Child/adolescent, Concussion, Ice hockey

## Abstract

**Background:**

KINARM end point robotic testing on a range of tasks evaluating sensory, motor and cognitive function in children/adolescents with no neurologic impairment has been shown to be reliable. The objective of this study was to determine whether differences in baseline performance on multiple robotic tasks could be identified between pediatric/adolescent ice hockey players (age range 10–14) with and without a history of concussion.

**Methods:**

Three hundred and eighty-five pediatric/adolescent ice hockey players (ages 10–14) completed robotic testing (94 with and 292 without a history of concussion). Five robotic tasks characterized sensorimotor and/or cognitive performance with assessment of reaching, position sense, bimanual motor function, visuospatial skills, attention and decision-making. Seventy-six performance parameters are reported across all tasks.

**Results:**

There were no significant differences in performance demonstrated between children with a history of concussion [median number of days since last concussion: 480 (range 8–3330)] and those without across all five tasks. Performance by the children with no history of concussion was used to identify parameter reference ranges that spanned 95 % of the group. All 76 parameter means from the concussion group fell within the normative reference ranges.

**Conclusions:**

There are no differences in sensorimotor and/or cognitive performance across multiple parameters using KINARM end point robotic testing in children/adolescents with or without a history of concussion.

## Background

The rate of child and adolescent participation in organized sport is high, which has significant health benefits related to regular exercise. However, youth sustain sport-related concussions, accounting for more than 15 % of all injuries in 9–16 year old players [1–3]. Concussion is a brain injury and has been defined as a complex pathophysiological process affecting the brain, induced by biomechanical forces [4]. In general, the majority (80–90 %) of concussions resolve in a short (7–10 days) period [5, 6]. Our understanding of the impact of concussion(s) on the brain is limited, however neuropsychological deficits have been observed in adults over a time span ranging from 24 h to 3 years [7–15]. Within the last decade research related to concussion in children and adolescents has rapidly expanded [16–18].

Of particular interest is the number of sport-related concussions sustained while playing ice hockey, which is popular in Canada and the USA with about 850,000 children playing in both countries [1, 2]. There is growing concern regarding the impact of concussion in this population [3, 16–22]. For example in Alberta, Canada, overall concussion injury rates [based on the number of injuries per 1000 player-hours (95 % confidence interval)] have been shown to range from 0.79 (0.55 to 1.13) to 2.73 (1.90 to 3.94) [21–23]. Researchers from London, Ontario, Canada examined a retrospective cohort of children/adolescents (<18 years of age) attending the emergency department who had sustained concussions (2006 to 2011). They demonstrated that 36 % of youth that sustained a sport-related concussion did so while playing ice hockey [24]. Evidence suggests that children and adolescents may be more susceptible to concussion, and may take longer to recover than adults [16–18, 25]. The impact of sport related concussion(s) on motor and cognitive processing in children, with respect to the effect on the developing brain, is poorly understood [26, 27].

The injury spectrum associated with concussion is broad, ranging from subtle or imperceptible to obvious changes in motor and/or cognitive performance, and very dependent on the developmental stage of the central nervous system (CNS) [28–31]. One of the primary reasons for the paucity of research related to the effect of concussion in children and adolescents is the lack of sensitive measurement tools that can identify impairments following concussion [32, 33]. Better diagnostic and prognostic tools are needed to address issues related to early diagnosis and management of concussion across the continuum of aging but particularly in children and adolescents. Maturation occurs at different rates across various domains within the CNS, ranging broadly from 18 years of age (reaching correction) to 30 (precision of number sense), which can complicate concussion evaluation in children and adolescents [34–36]. Researchers are beginning to examine the efficacy of different measurement tools used with adults among children and adolescents [37, 38].

Robotic technology has the potential for use as a clinical diagnostic assessment tool as it is ideal for objective, quantitative, rapid and automated assessment of neural function [39, 40]. Further, robots have often been used as treatment tools for individuals with brain damage [41–45]. The KINARM exoskeleton (BKIN Technologies Ltd, Ontario, Canada) is a robotic device that has been used to detect functional impairments across neurological domains in adults [40, 46–52]. Various tasks test visuomotor skills, proprioceptive function, rapid decision making, and executive function capabilities [46–52]. The KINARM end-point robot has been used to examine neurologic impairments in adult subjects post-concussion [52, 53]. The results from one study identified subjects with post-concussion syndrome (symptoms of the concussion that persist for weeks or months) had more abnormal scores than those without post-concussion syndrome [52, 54, 55]. There is evidence that the KINARM exoskeleton robot is reliable and sufficiently sensitive to use in adult stroke and moderate/severe brain injury populations [40, 46–51]. The KINARM end point robot also shows both relative reliability (intra-class correlation coefficients) and absolute reliability (Bland-Altman agreement) among healthy boys with no neurological impairment, who range in age from 10 to 14 [56].

The primary objective of the current study was to evaluate differences in KINARM end point robotic testing outcomes (sensorimotor and cognitive) between children/adolescent ice hockey players with and without a history of concussion. Comparisons in performance were made between those with a history of concussion [median number of days since last concussion: 480 (range 8–3330)] and those without. Reference ranges were determined for the robotic tests based on data from the controls (no history of concussion). Further, the reported history of concussion on subject performance was examined relative to the parameters of the five KINARM tasks.

## Methods

### Participants

**Fig. 1:**
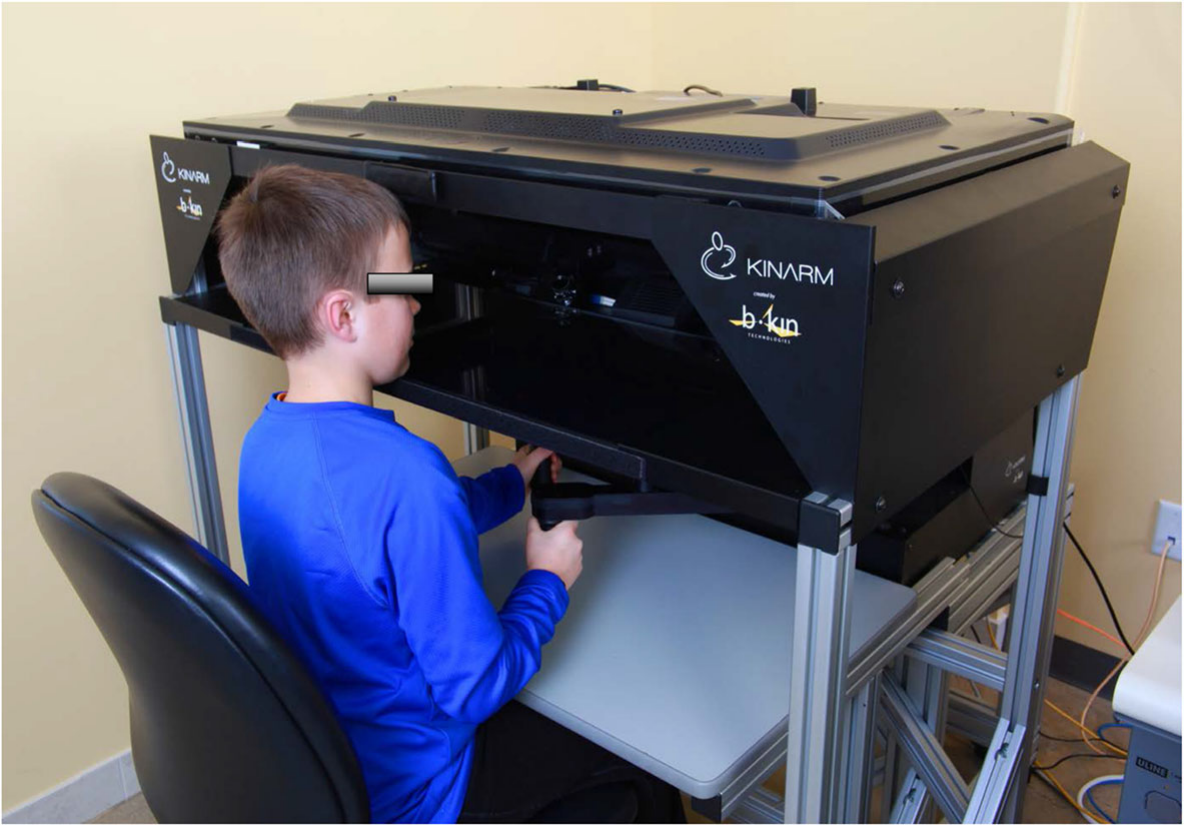
The KINARM end point robot. The virtual reality workstation projects targets onto a screen

The study is an observational cross-sectional design. Three hundred and eighty-five healthy children/adolescents (males and females) completed KINARM end point robot testing: 94 with a history of concussion and 292 without. Inclusion criteria for the present study were: ice-hockey players, ages 10–14, with no current neurological symptoms, and with and without a prior traumatically induced transient disturbance of the brain [57]. Subjects were excluded if they had a significant orthopedic injury (i.e. fracture) in the upper extremity, visual impairment (<20/50 corrected) or had sustained a concussion less than 5 days prior to testing. Concussion history was obtained by self-report through the use of a questionnaire (adapted from a similar questionnaire used in youth hockey studies previously conducted in this laboratory) that children completed, with the help of their parents [21]. Subjects were asked to record the number of previously sustained concussions (whether diagnosed by a physician) as well as the date and activity participating in at the time of the concussion. The most recent concussion referenced was used for the analysis in the current study. This was a sample of convenience. The Conjoint Health Research Ethics Board at the University of Calgary approved the study (Ethics ID number E24026). Prior to data collection, a parent or guardian provided signed consent for the subject to participate in all aspects of the study and the children provided assent. Parental consent was provided for the photograph in Fig. 1 to be included in the current publication.

### Robotic assessment

At the beginning of the 2013–2014 ice hockey season players from Pee Wee ice hockey teams (ages 10–14) completed KINARM end point robot testing at the University of Calgary. The robotic assessment was performed using the KINARM end point bimanual device (BKIN Technologies Ltd, Kingston, Ontario, Canada), which permits free movement of the upper extremities in the horizontal plane while seated (refer to Fig. 1). A virtual reality system displays visual targets such that they appear in the same plane as the arms. Subjects experience force feedback while grasping the robot handles when hitting targets during specific tasks. The protocol included assessments of reaching, position sense, bimanual motor function, visuospatial skills, attention, and decision-making [56].

The testing session lasted approximately 15 min, which included seating the subject at the robot, instructions and completion of the five tasks. Subjects were seated in a chair in front of the robot, asked to avoid slouching, and the robot height adjusted such that each child’s head rested on a location in the center of the visual field. Body position was kept constant across subjects. Subjects completed the following five tasks during each testing session: Visually Guided Reaching (VGR) on right and left, Arm Position Matching (APM) on right and left, Object Hit (OH), Object Hit and Avoid (OHA), and Trail Making B (TMB) with the dominant limb [56]. These tasks characterize sensorimotor and/or cognitive performance. Seventy-six parameters from the five tasks are presented in the paper.

### Experimental tasks

#### Visually guided reaching task

**Fig. 2:**
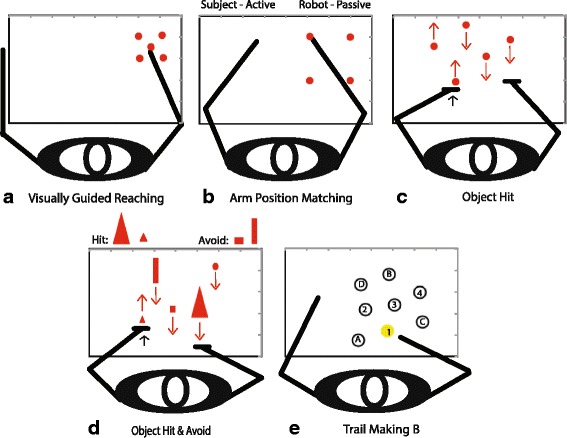
The five KINARM robot tasks used in the study. **a** Visually guided reaching with the right arm, **b** Arm position matching with the right arm, **c** Object hit, **d** Object hit and avoid, and **e**. Trail making B (not to scale, example of the alpha-numeric alternation) This figure has been modified from a previous paper [56]

This task provides a measure of upper extremity visuomotor capability (Fig. 2a) [56]. The robot handle is represented as a white dot (0.5-cm radius) on the display. The task targets are red circles, each with a 1.0-cm radius. Subjects reach out and back between the central and destination targets. Four red targets are 10 cm from the initial central target. Subjects are asked to move the white dot from the centre of one target to the centre of the next target that appears, as quickly and accurately as possible. All targets are located near the centre of the workspace for each arm. There are five blocks of trials, target location is randomized within a block and both the reach out and reach back trials are analyzed. This process is repeated forty times to explore the workspace and measure variability of the subject’s responses. Each subject completed the task twice, once with each arm; the dominant arm always preceded the non-dominant arm. Although not identical, the task used in the current work is similar to and uses metrics that were described earlier using the KINARM exoskeleton robot [46–48, 50, 56].

#### Arm position matching task

This task provides a measure of proprioceptive (position sense) capability (Fig. 2b) [56]. The robot moves one arm (passive arm) to one of four different target locations spaced at the corners of a square grid at 20 cm intervals in the X and Y directions. Movements are made with a bell-shaped velocity profile. Subjects are then instructed to actively move the opposite arm (active arm) to the mirror location of the passive arm in space. Subjects notified the examiner once the mirror-matched position was reached and the examiner advanced the robot to the next trial. The subject’s vision is blocked to ensure that any sensory information about limb position is from proprioceptive inputs. There are six blocks of trials, target location is randomized within a block and one trial for each target is completed within a block. The same target is never repeated sequentially. The task was completed twice with dominant arm being the active arm first followed by the non-dominant arm. A similar task has been used with the KINARM exoskeleton robot; to reduce time for the assessment of subjects four targets were used rather than nine [46–48, 50, 56].

#### Object hit task

This task is a rapid sensorimotor, decision and control test (Fig. 2c) [56]. It assesses the ability of a subject to select and engage motor actions with both hands over a range of speeds and a large workspace. Virtual paddles appear at the robot handles. Subjects are asked to use the paddles to hit balls that appear to fall from the top of the screen toward them. The robot produces a reactive force that mimics the actual force that would have been felt by the subject if these were real objects contacting a real paddle (perceived as having physical properties similar to a squash ball). As the task proceeds the balls move at greater speeds and appear more often, making the task more difficult as time progresses. Balls fall at random from ten bins that are spread equally across the workspace, 30 balls fall from each bin. A total of 300 balls are dropped during the task in just over one minute. A similar task has been used with older adults and the KINARM exoskeleton robot, except the version used in the current work used smaller paddles (2 cm rather than 5 cm), the balls moved faster and more balls could be present on the screen at a given time [49, 56].

#### Object hit and avoid

This task is similar to the OH task, but requires more cognitive control. Subjects must hit specific target objects while avoiding all others (Fig. 2d) [56]. The emphasis is on attention, rapid motor selection, and inhibition to avoid distractor targets. At the start of the task subjects are shown two target shapes out of a possible eight, they are instructed to memorize these as the only two shapes to hit during the task, and to avoid all other (6) distractor shapes. If distractors hit the subject’s paddles they pass through the paddles but there is no reactive force felt by the subject. This provides immediate and ongoing feedback to the subject that the object was a distracter and not a target. As with the preceding task, when targets are hit the robot produces a reactive force that mimics the actual force that would have been felt by the subject if these were real objects contacting a real paddle. A similar task has been used with older adults and the KINARM exoskeleton robot, except the version used in the current work used smaller paddles (2 cm rather than 5 cm), the objects moved slightly faster and more objects could be present on the screen at a given time [56].

#### Trail Making B task

This task is the second part of a cognitive test that evaluates executive function (e.g. visual attention and task switching) from the field of neuropsychology that is commonly used in the assessment of brain injury (Fig. 2e) [38, 56]. Participants trace through an alternating alpha-numeric sequence of targets 1-A-2-B for example, up to 13, for a total of 25 targets. A shortened version of the task, that has only five sequential targets preceded the full task to help familiarize subjects with the task. If the subject touches an incorrect target while moving through the sequence the preceding correct target will turn red and the subject must return to that target before continuing. The task pattern was randomly selected from eight possible patterns [Matlab (Mathworks, Natick, MA, USA)] for the TMB task [56, 58].

### Outcome measures

Parameters for each task have been previously developed to quantify task performance in healthy individuals and those with brain damage, thus behavioral attributes associated with the parameters are included in Table 1. In summary, the visually guided reaching task included 10 parameters: posture speed (m/s), reaction time (s), initial direction error (rad), initial distance ratio, initial speed ratio, speed maxima count, minimum maximum speed difference (m/s), movement time (s), path length ratio, and max speed (m/s). The arm position matching task included 12 parameters: variability X, Y, and XY (m), contraction/expansion ratio X, Y, and XY, shift X, Y, and XY (m), absolute error X, Y, and XY. The object hit task included 14 parameters: total hits, hits with left, hits with right, hand bias hits, miss bias, hand transition, hand selection overlap, median error, hand speed left and right (m/s), movement area left and right (m^2^), and movement area bias. Object hit and avoid include the same parameters as outlined for object hit (hand bias hits, hand speed bias, and movement area bias were removed from the primary analysis) with three additions: distractor hits left, right and total. Trail making task B included the following four parameters: total time (s), dwell time (s), time ratio and error count.

**Table 1:** Summary of the five KINARM robot tasks, including behavioral attributes and definitions of task parameters

Task	Parameter	Behavioral attribute	Definition
Visually guided reaching	Posture speed (m/s)	Upper Extremity Posture Control	Mean hand speed (when hand should be at rest).
	Reaction time (s)	Response to a Visual Stimulus	Difference in time between target illumination and movement onset.
	Initial direction error (rad)	Initial phase of the movement (Feed-forward control)	Angular deviation between a straight line (1) from the hand position at movement onset to the destination target and (2) from the hand position at movement onset to the hand position after the initial phase of movement.
	Initial distance ratio	Initial phase of the movement (Feed-forward control)	Ratio of the distance the hand travelled (1) during the subject’s initial phase of movement to (2) between movement onset and movement offset (or the end of the trial if the destination target is not reached).
	Initial speed ratio	Initial phase of the movement (Feed-forward control)	The maximum hand speed during the subject’s initial phase of movement/ the global hand speed maximum of the trial (Ratio).
	Speed maxima count	Corrective Movement following the initial motor response	Hand speed maxima between movement onset and offset (Total).
	Minimum maximum speed difference (m/s)	Corrective Movement following the initial motor response	Hand speed maxima minus minima.
	Movement time (s)	Entire Movement	Time elapsed from movement onset to end (Total).
	Path length ratio	Entire Movement	The distance travelled by the hand between the movement onset and movement offset/ the straight line distance between the starting and destination targets (Ratio).
	Max speed (m/s)	Entire Movement	Maximum hand speed (Global).
Arm position matching	Variability X (m)	Position sense	Mean value of the variability of the subject’s hand position (X direction).
	Variability Y (m)	Position sense	Mean value of the variability of the subject’s hand position (Y direction).
	Variability XY (m)	Position sense	RMS of X and Y variables.
	Contraction/expansion ratio X	Position sense	Ratio of the movement range in the x-direction (arm moved by the subject compared to the arm moved by the robot).
	Contraction/expansion ratio Y	Position sense	Ratio of the movement range in the y-direction.
	Contraction/expansion ratio XY	Position sense	Ratio of the range of area moved over.
	Shift X (m)	Position sense	Mean difference between the mirrored x-position of the arm moved by the subject and the x-position of the arm moved by the robot (+ lateral shift, − medial shift).
	Shift Y (m)	Position sense	Mean difference, as above, but in the y-direction.
	Shift XY (m)	Position sense	RMS of the X and Y shifts.
	Absolute Error X	Position sense	The mean absolute distance error in the X direction (All trials).
	Absolute Error Y	Position sense	The mean absolute distance error in the Y direction (All trials).
	Absolute Error XY	Position sense	The mean absolute distance error across all trials.
Object hit	Total hits	Performance (Global)	Balls hit off the screen in the opposite direction from it original path (Total).
	Hits with left	Performance (Global)	Balls hit with the left (L) hand (Total).
	Hit with right	Performance (Global)	Balls hit with the right (R) hand (Total).
	Hand bias hits	Performance (Motor)	Quantifies the hand that is used more often for hitting the balls (hand dominance).
	Miss bias	Performance (Spatial & Temporal)	Quantifies any bias of misses toward one side of the workspace or the other (x direction only).
	Hand transition	Performance (Spatial & Temporal)	Identifies where the subject’s preference for using one hand over the other switches in the workspace.
	Hand selection overlap	Performance (Motor)	Identifies how effective subjects are at using both hands and how often they overlap hands (i.e. hit balls with both the R and L hands in the same are of the work space).
	Median error	Performance (Spatial & Temporal)	Percentage of task completion when the subject made half their errors.
	Hand speed L (m/s)	Performance (Motor)	Mean L hand speed maintained throughout the task.
	Hand speed R (m/s)	Performance (Motor)	Mean R hand speed maintained throughout the task.
	Hand speed bias		The bias in hand speed between the hands (−1 to 1).
	Movement area L & R (m^2)	Performance (Motor)	Surface area the subject used with each hand during the task.
	Movement area bias	Performance (Motor)	The bias in movement area between hands (1 to 1).
Object hit & avoid	Parameters are the same as those identified for Object Hit, with three additions – see below. Note – Hand bias hits, Hand speed bias, and Movement area bias were removed from the primary analysis.		
	Distractor hits L	Performance (Global)	Distractor objects hit with L hand (Total).
	Distractor hits R	Performance (Global)	Distractor objects hit with the R hand (Total).
	Distractor hits total	Performance (Global)	Distractor objects the subject hit; reported as the % of total distracters dropped (Total).
Trail Making B	Total time (s)	Executive Function	Time from the targets being illuminated to touching the last target (Total).
	Dwell time (s)	Executive Function	Time spent with the hand feedback dot at the targets (Total).
	Time ratio		Time for targets 13–25/time for targets 1–12 (Ratio).
	Error count		Times an incorrect target was touched (Total).

Behavioral attributes and definitions of task parameters are outlined. The table has been modified from a previous paper [56]

### Data analysis

Performance by the group with no history of concussion was used to identify reference ranges for each parameter that spanned 95 % of the group [47]. Most of the time, the 95 % range was one-sided, reflecting the fact that abnormal values would be expected to be larger or smaller than the comparison sample (e.g. reaction time would be expected to be slower in individuals with concussion). Mean values of subjects with a history of concussion were calculated and then assessed to identify if they fell within the reference ranges.

All subjects and their data were included in the analysis as there were no missing data points. Individuals with a history of concussion [median number of days since last concussion: 480 (range 8–3330)] were further subdivided. Two separate grouping strategies are presented in an attempt to fully explore the relationship of timing since concussion to performance of those children with a history of concussion as related to the reference range developed from the performance of children with no history of concussion. In grouping A, the data from children with a history of concussion was divided into three equal terciles based on time since their last concussion to determine whether timeline since concussion would impact parameter performance. Terciles were chosen to ensure that an equivalent number of children were present in each of the three bins. The timelines associated with each tercile are as follows: T1 = 8–330 days (0–11 months), T2 = 331–990 days (11–33 months) and T3 = 991–3330 days (33–111 months). The time since their most recent concussion was obtained from a questionnaire (adapted from a similar questionnaire used in youth hockey studies previously conducted in this laboratory) that children filled out at home with parental input and submitted to the researchers at testing [21]. In grouping B, we were specifically interested in those cases that had been concussed in the last year. We divided the data according to five specific time points post-concussion: 3 weeks, 1, 3, 6 months, and 1 year.

Statistical analyses were performed in SPSS version 21 [59]. The significance level was set at alpha = 0.05. Since eight different patterns were randomly selected across subjects for the TMB task, a preliminary analysis was performed to ensure that parameter outcomes were not affected by TMB task version. Total time (s) was selected as the representative dependent variable in the TMB task analysis and was performed on the group of subjects with no history of concussion. Output from a one-way analysis of variance (ANOVA) showed no statistically significant difference in performance across the eight versions of the TMB tasks used during testing, F(7, 28) = 1.748, *p* = 0.098, thus parameters for each version of the TMB task were collapsed in order to perform the primary analysis for the TMB task.

The primary analysis in the current study included separate multivariate analyses of variance (MANOVA) for each of the five robot tasks, with right and left side analyzed separately for the Visually Guided Reaching and Arm Position Matching tasks. Those children/adolescents with and without a history of concussion were compared to determine differences in performance. Separate exploratory analyses were performed using MANOVA for each of the five robot tasks to evaluate if differences existed between age groups (Ages 10 and 11 versus Age group 12,13,14) and for each of the five robot tasks to evaluate if differences existed between Handedness groups (Right versus Left). As stated above right and left side were analyzed separately for the Visually Guided Reaching and Arm Position Matching tasks. One-way analysis of variance (ANOVA) was used to identify differences across individual parameters when significant group effects were found.

## Results

Characteristics of the subjects who took part in the study are found in Table 2. The primary analysis showed no significant difference in performance, based on parameter values, between those children/adolescents with and without a history of concussion across all five tasks (refer to Table 3). Table 4 presents a summary of the outcomes from the secondary exploratory analysis from separate MANOVAs that compared Age Groups and Handedness. The results from the MANOVA exploratory analysis showed that there was a difference in at least one of the variables between Age Groups, while the ANOVAs identified which parameters were significantly different: Visually Guided Reaching (R & L), Object Hit, Object Hit and Avoid, and TMB task. Furthermore, the MANOVA analysis also showed a difference in at least one of the variables related to Handedness, while the ANOVAs also identified which parameters were significantly different: Visually Guided Reach (L only), Arm Position Matching (R only), and Object Hit.

**Table 2:** Summary of the study population characteristics

	NO history of concussion	History of concussion
Subjects	*n* = 292	*n* = 93
Age – Years: mean (95 % confidence interval)	11.3 (11.2, 11.5)	11.5 (11.3, 11.6)
Age Range	10–14 years	10–12 years
Age group 1 (10,11): frequency (proportion)	*n* = 179 (61 %)	*n* = 46 (49 %)
Age group 2 (12,13,14): frequency (proportion)	*n* = 113 (39 %)	*n* = 47 (51 %)
Sex: frequency (proportion)	M = 272 (93 %)	M =90 (97 %)
	F = 20 (7 %)	F = 3 (3 %)
Weight (kg): mean (95 % confidence interval)	M = 43.1 (41.9, 44.3)	M = 43.4 (41.4, 45.4)
	F = 44.1 (40.4, 47.8)	F = 35.8 (27.6, 44.1)
Height (cm): mean (95 % confidence interval)	M = 151.2 (150.2, 152.2)	M = 151.4 (149.8, 153.0)
	F =151.3 (146.7, 155.9)	F = 145.5 (133.3, 157.6)
Dominant Hand (proportion)	R = 258 (88 %)	R = 81 (87 %)
Hand: frequency (proportion)	L = 34 (12 %)	L = 12 (13 %)

Subject characteristics with mean (95 % confidence intervals), range, and frequency (proportion) included

**Table 3:** Primary statistical analysis outcomes

Task	MANOVA
Visually guided reach	Right: F(10,375) = 0.481, *p* = 0.90
	Left: F(10,375) = 1.324, *p* = 0.215
Arm position matching	Right: F(12, 373) = 1.053, *p* = 0.399
	Left: F(12, 373) = 0.886, *p* = 0.562
Object hit	F(13, 371) = 1.217, *p* = 0.264
Object hit & avoid	F(12,373) = 1.094, *p* = 0.364
Trail Making B	F(4, 377) = 0.278, *p* = 0.892

Summary of MANOVA outputs comparing children/adolescents with and without a history of concussion across all five KINARM robot tasks

**Table 4:** Exploratory statistical analysis outcomes

KINARM robot tasks	Age group	Age group 10,11: mean (SD)	Age 12,13,14: mean (SD)	Handedness	Right: mean (SD)	Left: mean (SD)
Visually guided reach: right	F(10, 374) = 2.337, *p* = 0.011	NA	NA	F(10, 374) = 1.498, *p* = 0.138	NA	NA
Reaction time (s)	*p* = 0.018	0.282 (0.097)	0.264 (0.030)	NA	NA	NA
Initial direction error (rad)	*p* = 0.003	0.053 (0.024)	0.046 (0.021)	NA	NA	NA
Initial distance ratio	*p* = 0.001	0.853 (0.098)	0.885 (0.073)	NA	NA	NA
Initial speed ratio	*p* = 0.001	0.963 (0.040)	0.976 (0.029)	NA	NA	NA
Speed max count	*p* = 0.004	2.60 (0.460	2.46 (0.495)	NA	NA	NA
Movement time (s)	*p* < 0.001	1.111 (0.183)	1.044 (0.159)	NA	NA	NA
Max speed (m/s)	*p* = 0.002	0.254 (0.071)	0.277 (0.067)	NA	NA	NA
Visually guided reach: left	F(10, 374) = 3.189, *p* = 0.001	NA	NA	F(10, 374) = 3.139, *p* = 0.001	NA	NA
Reaction time (s)	*p* < 0.001	0.280 (0.037)	0.265 (0.031)	NA	NA	NA
Initial direction error (rad)	*p* = 0.003	0.883 (0.067)	0.901 (0.043)	*p* = 0.008	0.049 (0.016)	0.043 (0.015)
Movement time (s)	*p* = 0.006	1.069 (0.136)	1.031 (0.126)	NA	NA	NA
Max speed (m/s)	*p* = 0.006	0.269 (0.071)	0.290 (0.072)	NA	NA	NA
Speed max count	NA	NA	NA	*p* = 0.03	2.35 (0.35)	2.47 (0.42)
Path length ratio	NA	NA	NA	*p* = 0.037	1.19 (0.09)	1.16 (0.070
Arm position matching: right	F(12, 372) = 0.977, *p* = 0.470	NA	NA	F(12, 372) = 1.846, *p* = 0.040	NA	NA
Contraction/expansion ratio X	NA	NA	NA	*p* = 0.023	0.922 (0.280)	1.022 (0.270)
Contraction/expansion ratio Y	NA	NA	NA	*p* = 0.045	0.983 (0.102)	1.014 (0.074)
Contraction/expansion ratio XY	NA	NA	NA	*p* = 0.009	0.919 (0.309)	1.045 (0.299)
Shift Y	NA	NA	NA	*p* = 0.029	−0.008 (0.019)	−0.014 (0.013)
Arm position matching: left	F(12, 372) =1.603, *p* = 0.088	NA	NA	F(12, 372) = 0.916, *p* = 0.531	NA	NA
Object hit	F(13, 370) =3.657, *p* < 0.001	NA	NA	F(13,370) = 5.678, *p* < 0.001	NA	NA
Total hits	*p* < 0.001	152 (24)	167 (22)	NA	NA	NA
Hits with L	*p* < 0.001	70 (14)	77 (13)	*p* < 0.001	72 (13)	82 (16)
Hits with R	*p* < 0.001	82 (15)	90 (15)	*p* = 0.001	86 (15)	78 (16)
Median error	*p* = 0.001	58 (3)	59 (3)	NA	NA	NA
Hand speed L (m/s)	*p* = 0.005	0.229 (0.054)	0.254 (0.059)	NA	NA	NA
Hand speed R (m/s)	*p* = 0.037	0.255 (0.062)	0.235 (0.058)	NA	NA	NA
Hand speed bias	*p* = 0.001	0.052 (0.100)	−0.037 (0.110)	*p* < 0.001	0.052 (0.076)	−0.027 (0.092)
Movement area bias	*p* = 0.001	0.036 (0.101)	−0.024 (0.117)	NA	NA	NA
Hand bias hits	NA	NA	NA	*p* = 0.001	0.092 (0.093)	−0.027 (0.115)
Miss bias	NA	NA	NA	*p* = 0.004	−0.002 (0.032)	0.012 (0.029)
Hand transition	NA	NA	NA	*p* = 0.009	−0.017 (0.032)	−0.003 (0.037)
Hand speed L (m/s)	NA	NA	NA	*p* = 0.003	0.302 (0.069)	0.335 (0.077)
Movement area (m^2)	NA	NA	NA	*p* = 0.004	0.035 (0.077)	−0.001 (0.084)
Object hit & avoid	F(12, 372) =2.436, *p* = 0.005	NA	NA	F(12, 372) = 4.506, *p* = 0.127	NA	NA
Total hits	*p* < 0.001	102 (17)	111 (18)	NA	NA	NA
Hits with L	*p* < 0.001	47 (10)	52 (11)	NA	NA	NA
Hits with R	*p* < 0.001	55 (11)	59 (11)	NA	NA	NA
Median error	*p* = 0.029	59 (5)	60 (5)	NA	NA	NA
Hand speed L (m/s)	*p* = 0.014	0.226 (0.054)	0.240 (0.055)	NA	NA	NA
Trail making task B	F(4, 376) = 4.256, *p* = 0.002	NA	NA	F(4, 376) =0.711, *p* = 0.585	NA	NA
Test time (s)	*p* < 0.001	64 (21)	56 (17)	NA	NA	NA
Dwell time (s)	*p* < 0.001	32 (11)	28 (11)	NA	NA	NA

Secondary exploratory analysis using separate MANOVAs for 1) Age Groups (Age 10 & 11 versus Age 12, 13, & 14) and 2) Handedness (Right versus Left) across all five KINARM tasks; mean parameter values are presented for those which were found to be statistically significant (*NA* not applicable)

**Fig. 3:**
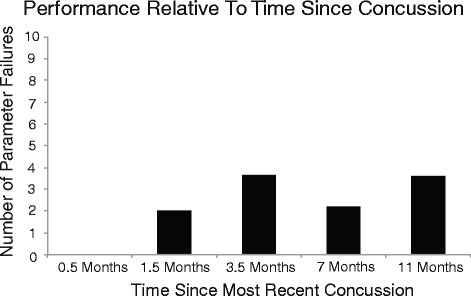
Title: Parameter failure versus time since most recent concussion. Represents the frequency that all subjects with a past history of concussion fell outside the 95 % range of controls relative to the time since their most recent concussion. The occurrence of this was rare considering at each time point there were 36 subjects and 76 parameters (i.e. The total potential parameter failure is 2736)

The reference ranges (based on performance from participants with no history of concussion) for parameters for all five KINARM end point robot tasks are presented in Table 5 and this includes identification of the direction of failure related to performance. Visual inspection was used to determine whether the mean values for parameters from children/adolescents with a history of concussion fell within the reference ranges (refer to Table 6). For grouping A of the data, the parameter mean values for all subjects in each of the terciles (time since most recent concussion) also fell within the established reference ranges (refer to Table 7). With respect to grouping B, due to the variability in the timelines among the children the mean and range about each of these time points are as follows: 3 weeks [0.5 months (8–21 days)], 1 month [1.5 months (30–60 days)], 3 months [3.5 months (90–120 days)], 6 months [7 months (150–270 days)], 1 year [11 months (300–360 days)]. An unequal number of children are present in each of the five bins: 0.5 months (2), 1.5 months (9), 3.5 months (3), 7 months (9), and 11 months (13). Refer to Fig. 3 for the frequency of parameter failures across the five time point bins. Figure 3 represents the frequency that all subjects with a past history of concussion fell outside the 95 % range of controls relative to the time since their most recent concussion. The occurrence of this was rare considering at each time point there were 36 subjects and 76 parameters (e.g. The total potential parameter failure is 2736).

**Table 5:** Parameter reference range established from healthy controls

Visually guided reach	Failure	Normative distribution of reference range: %	Reference range: R	Reference range: L
Posture speed (m/s)	1-sided: faster is abnormal	0–95	0.00074–0.00562	0.00105–0.00625
Reaction time (s)	1-sided: slower is abnormal	0–95	0.207–0.332	0.214–0.330
Initial direction error (rad)	1-sided: larger is abnormal	0–95	0.0197–0.0887	0.01972–0.0792
Initial distance ratio	1-sided: smaller is abnormal	5–100	0.6771–1	0.8258–1
Initial speed ratio	1-sided: smaller is abnormal	5–100	0.9044–1	0.9182–1
Speed maxima count	1-sided: larger is abnormal	0–95	1.5526–3.2000	1.525–2.974
Minimum maximum speed difference (m/s)	1-sided: larger is abnormal	0–95	0.00503–0.0330	0.00531–0.03428
Movement time (s)	1-sided: larger is abnormal	0–95	0.7665–1.388	0.694–1.297
Path length ratio	1-sided: larger is abnormal	0–95	1.0422–1.2892	1.0511–1.3345
Max speed (m/s)	1-sided: smaller is abnormal	5–100	0.16405–0.52206	0.1856–0.6092
Arm position matching	Failure	Normative distribution of reference range: %	Reference range: R	Reference range: L
Variability X (m)	1-sided: larger is abnormal	0–95	0.0139–0.0861	0.0163–0.1017
Variability Y (m)	1-sided: larger is abnormal	0–95	0.0058–0.0287	0.0075–0.0331
Variability XY (m)	1-sided: larger is abnormal	0–95	0.0178–0.08892	0.0189–0.1060
Contraction/expansion ratio X	2-sided: under or over shoot	2.5–97.5	0.2313–1.300	0.1979–1.3720
Contraction/expansion ratio Y	2-sided: under or over shoot	2.5–97.5	0.7658–1.1623	0.8136–1.1636
Contraction/expansion ratio XY	2-sided: under or over shoot	2.5–97.5	0.2489–1.4161	0.1601–1.4422
Shift X (m)	2-sided: smaller or larger	2.5–97.5	−0.1200–0.0596	−0.123–0.052
Shift Y (m)	2-sided: smaller or larger	2.5–97.5	−0.0377–0.02391	−0.03992–0.01962
Shift XY (m)	2-sided: smaller or larger	5–100	0.00866–0.29611	0.01205–0.2531
Absolute Error X	1-sided: larger is abnormal	0–95	0.0135–0.1655	0.0187–0.1659
Absolute Error Y	1-sided: larger is abnormal	0–95	0.0061–0.0372	0.0076–0.0399
Absolute Error XY	1-sided: larger is abnormal	0–95	0.02033–0.16927	0.0259–0.1698
Object hit	Failure	Normative distribution of reference range: %	Reference range:	NA
Total hits	1-sided: smaller is abnormal	5–100	120–216	NA
Hits with left	1-sided: smaller is abnormal	5–100	50–116	NA
Hit with right	1-sided: smaller is abnormal	5–100	62–118	NA
Hand bias hits	2-sided: negL vs posR	2.5–97.5	−0.1579–0.2814	NA
Miss bias	2-sided: L vs R side of workspace	2.5–97.5	−0.0556–0.0665	NA
Hand transition	2-sided: L vs R side of workspace	2.5–97.5	−0.0764–0.0504	NA
Hand selection overlap	1-sided: smaller is abnormal	5–100	0.0779–0.3009	NA
Median error	1-sided: larger is abnormal	0–95	50–63	NA
Hand speed L (m/s)	1-sided: slower is abnormal	5–100	0.2073–0.5849	NA
Hand speed R (m/s)	1-sided: slower is abnormal	5–100	0.2330–0.5848	NA
Hand speed bias	2-sided: L vs R	2.5–97.5	−0.1545–0.2024	NA
Movement area L (m^2)	1-sided: smaller is abnormal	5–100	0.0871–0.2021	NA
Movement area R (m^2)	1-sided: smaller is abnormal	5–100	0.0951–0.2155	NA
Movement area bias	2-sided: L vs R	2.5–97.5	−0.1163–0.3828	NA
Object hit & avoid	Failure	Normative distribution of reference range: %	Reference range:	NA
Total hits	1-sided: smaller is abnormal	5–100	77–159	NA
Hits with left	1-sided: smaller is abnormal	5–100	31–74	NA
Hit with right	1-sided: smaller is abnormal	5–100	39–88	NA
Miss bias	2-sided: L vs R side of workspace	2.5–97.5	−0.0667–0.0664	NA
Hand transition	2-sided: L vs R side of workspace	2.5–97.5	−0.0923–0.0528	NA
Hand selection overlap	1-sided: smaller is abnormal	5–100	0.0430–0.2344	NA
Median error	1-sided: larger is abnormal	0–95	43–67	NA
Hand speed L (m/s)	1-sided: slower is abnormal	5–100	0.1469–0.4088	NA
Hand speed R (m/s)	1-sided: slower is abnormal	5–100	0.1506–0.4443	NA
Movement area L (m^2)	1-sided: smaller is abnormal	5–100	0.0797–0.1933	NA
Movement area R (m^2)	1-sided: smaller is abnormal	5–100	0.0866–0.2112	NA
Distractor hits L	1-sided: larger is abnormal	0–95	0–16	NA
Distractor hits R	1-sided: larger is abnormal	0–95	0–16	NA
Distractor hits total	1-sided: larger is abnormal	0–95	0–31	NA
Trail Making B	Failure	Normative distribution of reference range: %	Reference range:	NA
Total time (s)	1-sided: larger is abnormal	0–95	24–92	NA
Dwell time (s)	1-sided: larger is abnormal	0–95	9–49	NA
Time ratio	1-sided: larger is abnormal	0–95	0.3551–1.7837	NA
Error count	1-sided: larger is abnormal	0–95	0–6	NA

Summary of the healthy control reference range for parameters from the five KINARM robot tasks; includes identification of failure related to performance (*NA* not applicable)

**Table 6:** Parameter reference range for healthy controls versus children with concussion history

Visually guided reach	Reference range: R	History of concussion parameter means: R (Std)	Reference range: L	History of concussion parameter means: L (Std)
Posture speed (m/s)	0.00074–0.00562	0.003 (0.001)	0.00105–0.00625	0.004 (0.002)
Reaction time (s)	0.207–0.332	0.265 (0.031)	0.214–0.330	0.269 (0.035)
Initial direction error (rad)	0.0197–0.0887	0.048 (0.019)	0.01972–0.0792	0.047 (0.014)
Initial distance ratio	0.6771–1	0.867 (0.09)	0.8258–1	0.885 (0.076)
Initial speed ratio	0.9044–1	0.969 (0.029)	0.9182–1	0.972 (0.031)
Speed maxima count	1.5526–3.2000	2.550 (0.487)	1.525–2.974	2.374 (0.380)
Minimum maximum speed difference (m/s)	0.00503–0.0330	0.017 (0.008)	0.00531–0.03428	0.018 (0.009)
Movement time (s)	0.7665–1.388	1.097 (0.192)	0.694–1.297	1.064 (0.134)
Path length ratio	1.0422–1.2892	1.152 (0.071)	1.0511–1.3345	1.181 (0.095)
Max speed (m/s)	0.16405–0.52206	0.258 (0.074)	0.1856–0.6092	0.269 (0.080)
Arm position matching	Reference range	History of concussion parameter means: R (Std)	Reference range	History of concussion parameter means: L (Std)
Variability X (m)	0.0139–0.0861	0.047(0.028)	0.0163–0.1017	0.042(0.027)
Variability Y (m)	0.0058–0.0287	0.019(0.010)	0.0075–0.0331	0.017(0.008)
Variability XY (m)	0.0178–0.08892	0.052 (0.028)	0.0189–0.1060	0.045 (0.027)
Contraction/expansion ratio X	0.2313–1.300	0.913(0.312)	0.1979–1.3720	0.952(0.233)
Contraction/expansion ratio Y	0.7658–1.1623	0.983(0.130)	0.8136–1.1636	0.976(0.119)
Contraction/expansion ratio XY	0.2489–1.4161	0.918 (0.353)	0.1601–1.4422	0.944 (0.278)
Shift X (m)	−0.1200–0.0596	−0.030(0.059)	−0.123–0.052	−0.022(0.067)
Shift Y (m)	−0.0377–0.02391	−0.006(0.022)	−0.03992–0.01962	−0.009(0.018)
Shift XY (m)	0.00866–0.29611	0.052 (0.046)	0.01205–0.2531	0.050 (0.053)
Absolute Error X	0.0135–0.1655	0.067(0.040)	0.0187–0.1659	0.063(0.059)
Absolute Error Y	0.0061–0.0372	0.024(0.015)	0.0076–0.0399	0.022(0.016)
Absolute Error XY	0.02033–0.16927	0.076 (0.050)	0.0259–0.1698	0.071 (0.060)
Object hit	Reference range	History of concussion parameter means (Std)	NA	NA
Total hits	120–216	160 (27)	NA	NA
Hits with left	50–116	72 (14)	NA	NA
Hit with right	62–118	88 (18)	NA	NA
Hand bias hits	−0.1579–0.2814	0.096 (0.107)	NA	NA
Miss bias	−0.0556–0.0665	−0.006 (0.034)	NA	NA
Hand transition	−0.0764–0.0504	−0.016 (0.033)	NA	NA
Hand selection overlap	0.0779–0.3009	0.151 (0.042)	NA	NA
Median error	50–63	59 (3)	NA	NA
Hand speed L (m/s)	0.2073–0.5849	0.299 (0.069)	NA	NA
Hand speed R (m/s)	0.2330–0.5848	0.335 (0.086)	NA	NA
Hand speed bias	−0.1545–0.2024	0.052 (0.82)	NA	NA
Movement area L (m^2)	0.0871–0.2021	0.127 (0.029)	NA	NA
Movement area R (m^2)	0.0951–0.2155	0.139 (0.031)	NA	NA
Movement area bias	−0.1163–0.3828	0.044 (0.076)	NA	NA
Object hit & avoid	Reference range	History of concussion parameter means (Std)	NA	NA
Total hits	77–159	108 (19)	NA	NA
Hits with left	31–74	51 (10)	NA	NA
Hit with right	39–88	57 (12)	NA	NA
Miss bias	−0.0667–0.0664	0.006 (0.033)	NA	NA
Hand transition	−0.0923–0.0528	−0.020 (0.037)	NA	NA
Hand selection overlap	0.0430–0.2344	0.104 (0.039)	NA	NA
Median error	43–67	60 (6)	NA	NA
Hand speed L (m/s)	0.1469–0.4088	0.236 (0.053)	NA	NA
Hand speed R (m/s)	0.1506–0.4443	0.253 (0.066)	NA	NA
Movement area L (m^2)	0.0797–0.1933	0.120 (0.028)	NA	NA
Movement area R (m^2)	0.0866–0.2112	0.125 (0.029)	NA	NA
Distractor hits L	0–16	7 (4)	NA	NA
Distractor hits R	0–16	7 (5)	NA	NA
Distractor hits total	0–31	14 (9)	NA	NA
Trail Making B	Reference range	History of concussion parameter means (Std)	NA	NA
Total time (s)	24–92	60 (21)	NA	NA
Dwell time (s)	9–49	30 (11)	NA	NA
Time ratio	0.3551–1.7837	1.1 (0.43)	NA	NA
Error count	0–6	2 (2)	NA	NA

Summary of the healthy control reference range for parameters from the five KINARM robot tasks compared to parameter means from children/adolescents with a history of concussion (*NA* not applicable)

**Table 7:** Parameter reference range versus terciles based on time since last concussion

Visually guided reach	Reference range: R	Tercile 1: 0–11 months	Tercile 2: 11–33 months	Tercile 3: 33–111 months	Reference range: L	Tercile 1: 0–11 months	Tercile 2: 11–33 months	Tercile 3: 33–111 months
		Mean (Std)	Mean (Std)	Mean (Std)		Mean (Std)	Mean (Std)	Mean (Std)
Posture speed (m/s)	0.00074–0.00562	0.003(0.001)	0.003(0.002)	0.003(0.001)	0.00105–0.00625	0.003(0.002)	0.004(0.002)	0.003(0.001)
Reaction time (s)	0.207–0.332	0.257(0.030)	0.263(0.023)	0.275(0.038)	0.214–0.330	0.257(0.038)	0.278(0.037)	0.272(0.027)
Initial direction error (rad)	0.0197–0.0887	0.046(0.020)	0.050(0.017)	0.049(0.020)	0.01972–0.0792	0.044(0.013)	0.048(0.013)	0.049(0.015)
Initial distance ratio	0.6771–1	0.870(0.053)	0.868(0.106)	0.861(0.097)	0.8258–1	0.895(0.063)	0.887(0.062)	0.872(0.095)
Initial speed ratio	0.9044–1	0.966(0.031)	0.977(0.024)	0.965(0.031)	0.9182–1	0.973(0.025)	0.973(0.036)	0.971(0.031)
Speed maxima count	1.5526–3.2000	2.589(0.348)	2.469(0.555)	2.612(0.512)	1.525–2.974	2.410(0.379)	2.280(0.352)	2.431(0.391)
Minimum maximum speed difference (m/s)	0.00503–0.0330	0.018(0.008)	0.017(0.007)	0.016(0.009)	0.00531–0.03428	0.018(0.11)	0.019(0.008)	0.017(0.008)
Movement time (s)	0.7665–1.388	1.088(0.172)	1.082(0.181)	1.119(0.220)	0.694–1.297	1.056(0.104)	1.039(0.141)	1.093(0.151)
Path length ratio	1.0422–1.2892	1.164(0.072)	1.154(0.058)	1.141(0.079)	1.0511–1.3345	1.184(0.117)	1.186(0.074)	1.174(0.088)
Max speed (m/s)	0.16405–0.52206	0.272(0.079)	0.257(0.060)	0.247(0.079)	0.1856–0.6092	0.283(0.105)	0.270(0.059)	0.257(0.068)
Arm position matching	Reference range: R	Tercile 1: 0–11 months	Tercile 2:11–33 months	Tercile 3:33–111 months	Reference range: L	Tercile 1: 0–11 months	Tercile 2:11–33 months	Tercile 3:33–111 months
		Mean (Std)	Mean (Std)	Mean (Std)		Mean (Std)	Mean (Std)	Mean (Std)
Variability X (m)	0.0139–0.0861	0.043(0.024)	0.053(0.033)	0.046(0.023)	0.0163–0.1017	0.043(0.024)	0.038(0.017)	0.044(0.035)
Variability Y (m)	0.0058–0.0287	0.020(0.016)	0.018(0.007)	0.017(0.004)	0.0075–0.0331	0.018(0.012)	0.016(0.005)	0.017(0.006)
Variability XY (m)	0.0178–0.08892	0.048(0.027)	0.057(0.033)	0.049(0.023)	0.0189–0.1060	0.047(0.026)	0.041(0.070)	0.048(0.035)
Contraction/expansion ratio X	0.2313–1.300	0.974(0.273)	0.859(0.297)	0.900(0.349)	0.1979–1.3720	0.972(0.262)	0.951(0.235)	0.930(0.194)
Contraction/expansion ratio Y	0.7658–1.1623	0.977(0.187)	0.970(0.092)	1.001(0.070)	0.8136–1.1636	0.961(0.159)	0.965(0.099)	0.992(0.086)
Contraction/expansion ratio XY	0.2489–1.4161	0.986(0.333)	0.849(0.327)	0.914(0.384)	0.1601–1.4422	0.962(0.303)	0.927(0.283)	0.935(0.242)
Shift X (m)	−0.1200–0.0596	−0.020(0.052)	−0.025(0.059)	−0.044(0.065)	−0.123–0.052	−0.0001(0.064)	−0.029(0.067)	−0.036(0.064)
Shift Y (m)	−0.0377–0.02391	−0.005(0.023)	−0.003(0.024)	−0.008(0.018)	−0.03992–0.01962	−0.013(0.019)	−0.011(0.022)	−0.006(0.012)
Shift XY (m)	0.00866–0.29611	0.048(0.036)	0.050(0.046)	0.059(0.055)	0.01205–0.2531	0.052(0.043)	0.050(0.058)	0.048(0.060)
Absolute Error X	0.0135–0.1655	0.057(0.034)	0.070(0.053)	0.075(0.056)	0.0187–0.1659	0.060(0.046)	0.065(0.065)	0.064(0.064)
Absolute Error Y	0.0061–0.0372	0.026(0.020)	0.025(0.013)	0.022(0.010)	0.0076–0.0399	0.025(0.022)	0.024(0.015)	0.019(0.007)
Absolute Error XY	0.02033–0.16927	0.068(0.039)	0.078(0.053)	0.083(0.054)	0.0259–0.1698	0.070(0.052)	0.074(0.064)	0.071(0.063)
Object hit	Reference range:	Tercile 1: 0–11 months	Tercile 2:11–33 months	Tercile 3:33–111 months	NA	NA	NA	NA
		Mean (Std)	Mean (Std)	Mean (Std)				
Total hits	120–216	164(25)	158(26)	157(28)	NA	NA	NA	NA
Hits with left	50–116	73(15)	73(14)	70(14)	NA	NA	NA	NA
Hit with right	62–118	91(16)	86(18)	86(17)	NA	NA	NA	NA
Hand bias hits	−0.1579–0.2814	0.109(0.110)	0.073(0.122)	0.099(0.089)	NA	NA	NA	NA
Miss bias	−0.0556–0.0665	0.005(0.033)	−0.007(0.033)	−0.013(0.034)	NA	NA	NA	NA
Hand transition	−0.0764–0.0504	−0.026(0.034)	−0.011(0.035)	−0.011(0.029)	NA	NA	NA	NA
Hand selection overlap	0.0779–0.3009	0.151(0.031)	0.166(0.042)	0.133(0.044)	NA	NA	NA	NA
Median error	50–63	59(3)	58(3)	58(2)	NA	NA	NA	NA
Hand speed L (m/s)	0.2073–0.5849	0.304(0.062)	0.315(0.071)	0.280(0.070)	NA	NA	NA	NA
Hand speed R (m/s)	0.2330–0.5848	0.352(0.066)	0.388(0.094)	0.314(0.092)	NA	NA	NA	NA
Hand speed bias	−0.1545–0.2024	0.076(0.065)	0.027(0.085)	0.049(0.088)	NA	NA	NA	NA
Movement area L (m^2)	0.0871–0.2021	0.135(0.026)	0.132(0.030)	0.114(0.026)	NA	NA	NA	NA
Movement area R (m^2)	0.0951–0.2155	0.150(0.026)	0.140(0.032)	0.122(0.031)	NA	NA	NA	NA
Movement area bias	−0.1163–0.3828	0.052(0.075)	0.030(0.073)	0.050(0.080)	NA	NA	NA	NA
Object hit & avoid	Reference range:	Tercile 1: 0–11 months	Tercile 2: 11–33 months	Tercile 3: 33–111 months	NA	NA	NA	NA
		Mean (Std)	Mean (Std)	Mean (Std)				
Total hits	77–159	110(17)	108(20)	104(19)	NA	NA	NA	NA
Hits with left	31–74	50(10)	52(11)	49(10)	NA	NA	NA	NA
Hit with right	39–88	59(10)	56(12)	55(11)	NA	NA	NA	NA
Miss bias	−0.0667–0.0664	0.010(0.035)	0.005(0.034)	0.005(0.030)	NA	NA	NA	NA
Hand transition	−0.0923–0.0528	−0.028(0.037)	−0.012(0.038)	−0.020(0.036)	NA	NA	NA	NA
Hand selection overlap	0.0430–0.2344	0.106(0.040)	0.109(0.041)	0.098(0.035)	NA	NA	NA	NA
Median error	43–67	60(6)	60(8)	60(4)	NA	NA	NA	NA
Hand speed L (m/s)	0.1469–0.4088	0.244(0.050)	0.244(0.059)	0.223(0.050)	NA	NA	NA	NA
Hand speed R (m/s)	0.1506–0.4443	0.264(0.060)	0.256(0.073)	0.241(0.066)	NA	NA	NA	NA
Movement area L (m^2)	0.0797–0.1933	0.129(0.022)	0.123(0.029)	0.109(0.028)	NA	NA	NA	NA
Movement area R (m^2)	0.0866–0.2112	0.136(0.023)	0.123(0.030)	0.116(0.027)	NA	NA	NA	NA
Distractor hits L	0–16	6(4)	8(4)	7(4)	NA	NA	NA	NA
Distractor hits R	0–16	6(5)	8(5)	8(4)	NA	NA	NA	NA
Distractor hits total	0–31	12(8)	16(9)	15(8)	NA	NA	NA	NA
Trail Making B	Reference range:	Tercile 1: 0–11 months	Tercile 2:11–33 months	Tercile 3:33–111 months	NA	NA	NA	NA
		Mean (Std)	Mean (Std)	Mean (Std)				
Total time (s)	24–92	59(12)	57(28)	65(19)	NA	NA	NA	NA
Dwell time (s)	9–49	32(10)	25(10)	33(12)	NA	NA	NA	NA
Time ratio	0.3551–1.7837	1.148(0.524)	1.125(0.413)	0.994(0.301)	NA	NA	NA	NA
Error count	0–6	2(2)	3(3)	2(3)	NA	NA	NA	NA

Healthy control reference range versus terciles for grouping A, based on time since last concussion, across parameters from the five KINARM robot tasks (*NA* not applicable)

## Discussion

The main purpose of this study was to compare performance between children/adolescents with and without a history of concussion across five different robotic tasks, variations of which have previously been used to measure neurologic deficits in individuals with a variety of types of brain damage [40, 44, 46–51]. These tasks are designed to assess reaching, position sense, bimanual motor function, visuospatial skills, attention, and decision-making. The key finding is that the history of concussion group showed no difference in performance as compared to those subjects who reported no history of concussion.

The KINARM robot has specifically been used to evaluate Visually Guided Reaching and Arm Position Matching tasks in adults with mild to severe traumatic brain injury [47, 52]. In the current study, it is entirely possible that any neural impairment associated with concussion was resolved by the time the individuals were examined. In an attempt to address this issue subjects with a history of concussion were subdivided into two separate groupings (A and B) based on time since concussion. In the groups we examined we could find little to no difference in the presence of impairments on robotic testing based on timing.

Another consideration is that the impact of concussion on the elements of the nervous system examined in this study, if persistent, was so small that it fell well within the reference range recorded from individuals without a history of concussion. A limitation of the current study is the fact that individual subjects were not examined, only group effects. It could be that impairments were present on an individual basis, yet insufficient to cause a group effect. If a majority of the individuals with a history of concussion are truly symptom free this will mask the influence of the few individuals with real problems. Another limitation of the study is the fact that concussion history was obtained through self-report (children and parents). The accuracy of self-reported data on medical history is influenced by several factors such as the patient’s knowledge and understanding of the relevant information, ability to recall it, and willingness to report it [59]. Although the literature suggests there are problems with the reliability of self-report, self report measures are preferred as they are cost-effective and time efficient relative to physical examinations and lab testing particularly in large study groups [60–64]. In the current study there was simply no other way to conduct the study.

Another limitation of the study is that it is cross-sectional in design. Due to the nature of the fact that children were at various points from their concussion, this effectively increases the heterogeneity in our study. Prospective studies will be helpful moving forward. In this design, individuals can be evaluated before and after concussion and act as their own control. The heterogeneous nature of maturation within the central nervous system in this population however may result in increased levels of variability [65].

Due to the fact that, in many cases, there was a significant time lag between the most recent concussion and when we conducted the study, one could argue that this introduced a recall bias. However, this would be an equivalent issue in both groups as recall bias is non-differential, misclassification related to recall can occur in either group. It could also be argued that individuals in the no history of concussion group could be misclassified due to an undiagnosed concussion which has been raised in the literature related to impact sports such as ice-hockey and football [66].

Despite having a measurement tool that is reliable, accurate and precise, as mentioned previously, there can still be substantial variability across normal human behavior. This type of variability is something that can be seen in many biomarkers used in medicine and is simply one of the limitations of any tool that relies on a normative reference range [67]. One subject’s “normal” may represent a slight impairment for another individual. It may explain those “parameter failures” identified in subjects evaluated using grouping B. This could be dealt with by baseline testing all athletes and having individuals return when they sustain a concussion. In general children and adolescents have been shown to take longer to recover following concussion than their adult counterpart’s [15, 17].

The alternatives currently available to measure neurologic impairment following brain injury are prone to rely on observer-based ordinal scales or symptom questionnaires, each of which has their own set of limitations. For example, clinical symptom ratings are the foundation of most concussion management protocols [66]. However, many factors can affect symptom reporting in youth and adult athletes [68]. Symptom reporting can be influenced by a number of clinical, demographic and methodological variables. For example, post-concussion-like symptoms are nonspecific and are reported by uninjured athletes, healthy community-dwelling adults, and people with chronic pain [69–73]. In university settings, women have been shown to identify more symptoms on baseline testing than men [74]. In one study a subgroup of the study population reported no increase in symptoms post-concussion even though degradation in cognitive performance was identified on cognitive (ImPACT) testing [75].

Lastly, one might consider that concussion does not impact performance on the five tasks used in the present study. Preliminary evidence in elite athletes (mean age: 21 years) would suggest otherwise [53]. That study made comparisons between athletes at baseline and then again <72 h post-concussion using the same tasks used in the present study [54]. Over a period of days and weeks, those athletes returned to baseline [69]. In another study in adults with mild traumatic brain injury, conducted in the Emergency room in a Cincinnati (Ohio) hospital, four or more abnormal parameter scores were identified related to the Arm Position Matching task when compared to previously published normative reference data [47, 52]. Given the above findings we argue that, within the range of the neurologic impairments assessed these children have no persistent significant effects of their concussion.

The results from the exploratory analysis suggested a significant difference in performance between age groups in the following tasks: Visually Guided Reaching (for both the right and left upper extremity), Object Hit, Object Hit and Avoid, and Trail Making B tasks. The exploratory analysis also suggested a significant difference between upper extremity dominance (handedness) for the following: Visually Guided Reaching (left upper extremity) and Object Hit tasks. Age groups and handedness would be better suited to be covariates as part of multivariate analyses of variance, however the size of the current study population does not support this form of statistical analysis and is a limitation of the current study. The non-significant findings should not be equivocally interpreted as there being no difference at all. Given the small sample size, the study may have been underpowered to find a true significant difference. Another potential limitation of the present study is the issue of recall bias, in that subjects may have incorrectly reported a history of concussion.

## Conclusion

The current study presents reference ranges for parameters associated with five KINARM robotic tasks that assess reaching, position sense, bimanual motor function, visuospatial skills, attention, and decision-making. When children/adolescents with and without a history of concussion are compared, no differences in parameter performance were detected.

## Abbreviations

ANOVA: Analysis of variance
APM: Arm position matching
L: Left
MANOVA: Multivariate analysis of variance
OH: Object hit
OHA: Object hit and avoid
R: Right
TMB: Trail Making B
VGH: Visually guided reaching
